# Supplementary material for: Mapping the evolution of fertility support policies in China: A content and instrumental analysis
Source: PLoS One. 2025 Oct 9;20(10):e0332137. doi: 10.1371/journal.pone.0332137 (PMC12510515; doi:10.1371/journal.pone.0332137)
Supplement: S1 Appendix — (ZIP) [file pone.0332137.s001.zip › S1 Appendix. 226 original policy documents/55-国务院办公厅关于全面推进生育保险和职工基本医疗保险合并实施的意见.doc]

国务院办公厅关于全面推进生育保险和职工基本医疗保险合并实施的意见

**发布部门：** [国务院办公厅](https://www.pkulaw.com/law/chl?Aggs.IssueDepartment=601) [机构沿革](javascript:void(0)) [C:\email\index](../../../../C:/email/index)

**发文字号：**国办发〔2019〕10号

**发布日期：**2019.03.06

**实施日期：**2019.03.06

**时效性：** [现行有效](https://www.pkulaw.com/law/chl?Aggs.TimelinessDic=01)

**效力级别：** [国务院规范性文件](https://www.pkulaw.com/law/chl?Aggs.EffectivenessDic=XC0203)

**法规类别：** [保险综合规定](https://www.pkulaw.com/law/chl?Aggs.Category=04402)

国务院办公厅关于全面推进生育保险和职工基本医疗保险合并实施的意见
（国办发〔2019〕10号）

各省、自治区、直辖市人民政府，国务院各部委、各直属机构：
　　全面推进生育保险和职工基本医疗保险（以下统称两项保险）合并实施，是保障职工社会保险待遇、增强基金共济能力、提升经办服务水平的重要举措。根据《[中华人民共和国社会保险法](https://www.pkulaw.com/chl/4449c7967540e761bdfb.html)》有关规定，经国务院同意，现就两项保险合并实施提出以下意见。
　　一、指导思想
　　以习近平新时代中国特色社会主义思想为指导，全面贯彻党的十九大和十九届二中、三中全会精神，认真落实党中央、国务院决策部署，统筹推进“五位一体”总体布局和协调推进“四个全面”战略布局，坚持以人民为中心，牢固树立新发展理念，遵循保留险种、保障待遇、统一管理、降低成本的总体思路，推进两项保险合并实施，实现参保同步登记、基金合并运行、征缴管理一致、监督管理统一、经办服务一体化。通过整合两项保险基金及管理资源，强化基金共济能力，提升管理综合效能，降低管理运行成本，建立适应我国经济发展水平、优化保险管理资源、实现两项保险长期稳定可持续发展的制度体系和运行机制。

　　二、主要政策
　　（一）统一参保登记。参加职工基本医疗保险的在职职工同步参加生育保险。实施过程中要完善参保范围，结合全民参保登记计划摸清底数，促进实现应保尽保。
　　（二）统一基金征缴和管理。生育保险基金并入职工基本医疗保险基金，统一征缴，统筹层次一致。按照用人单位参加生育保险和职工基本医疗保险的缴费比例之和确定新的用人单位职工基本医疗保险费率，个人不缴纳生育保险费。同时，根据职工基本医疗保险基金支出情况和生育待遇的需求，按照收支平衡的原则，建立费率确定和调整机制。
　　职工基本医疗保险基金严格执行[社会保险基金财务制度](https://www.pkulaw.com/chl/e4e06e49beb752e2bdfb.html)，不再单列生育保险基金收入，在职工基本医疗保险统筹基金待遇支出中设置生育待遇支出项目。探索建立健全基金风险预警机制，坚持基金运行情况公开，加强内部控制，强化基金行政监督和社会监督，确保基金安全运行。
　　（三）统一医疗服务管理。两项保险合并实施后实行统一定点医疗服务管理。医疗保险经办机构与定点医疗机构签订相关医疗服务协议时，要将生育医疗服务有关要求和指标增加到协议内容中，并充分利用协议管理，强化对生育医疗服务的监控。执行基本医疗保险、工伤保险、生育保险药品目录以及基本医疗保险诊疗项目和医疗服务设施范围。
　　促进生育医疗服务行为规范。将生育医疗费用纳入医保支付方式改革范围，推动住院分娩等医疗费用按病种、产前检查按人头等方式付费。生育医疗费用原则上实行医疗保险经办机构与定点医疗机构直接结算。充分利用医保智能监控系统，强化监控和审核，控制生育医疗费用不合理增长。
　　（四）统一经办和信息服务。两项保险合并实施后，要统一经办管理，规范经办流程。经办管理统一由基本医疗保险经办机构负责，经费列入同级财政预算。充分利用医疗保险信息系统平台，实行信息系统一体化运行。原有生育保险医疗费用结算平台可暂时保留，待条件成熟后并入医疗保险结算平台。完善统计信息系统，确保及时全面准确反映生育保险基金运行、待遇享受人员、待遇支付等方面情况。
　　（五）确保职工生育期间的生育保险待遇不变。生育保险待遇包括《[中华人民共和国社会保险法](https://www.pkulaw.com/chl/4449c7967540e761bdfb.html)》规定的生育医疗费用和生育津贴，所需资金从职工基本医疗保险基金中支付。生育津贴支付期限按照《[女职工劳动保护特别规定](https://www.pkulaw.com/chl/cdf82292dbb62ee9bdfb.html)》等法律法规规定的产假期限执行。
　　（六）确保制度可持续。各地要通过整合两项保险基金增强基金统筹共济能力；研判当前和今后人口形势对生育保险支出的影响，增强风险防范意识和制度保障能力；按照“尽力而为、量力而行”的原则，坚持从实际出发，从保障基本权益做起，合理引导预期；跟踪分析合并实施后基金运行情况和支出结构，完善生育保险监测指标；根据生育保险支出需求，建立费率动态调整机制，防范风险转嫁，实现制度可持续发展。

　　三、保障措施
　　（一）加强组织领导。两项保险合并实施是党中央、国务院作出的一项重要部署，也是推动建立更加公平更可持续社会保障制度的重要内容。各省（自治区、直辖市）要高度重视，加强领导，有序推进相关工作。国家医保局、财政部、国家卫生健康委要会同有关方面加强工作指导，及时研究解决工作中遇到的困难和问题，重要情况及时报告国务院。
　　（二）精心组织实施。各地要高度重视两项保险合并实施工作，按照本意见要求，根据当地生育保险和职工基本医疗保险参保人群差异、基金支付能力、待遇保障水平等因素进行综合分析和研究，周密组织实施，确保参保人员相关待遇不降低、基金收支平衡，保证平稳过渡。各省（自治区、直辖市）要加强工作部署，督促指导各统筹地区加快落实，2019年底前实现两项保险合并实施。
　　（三）加强政策宣传。各统筹地区要坚持正确的舆论导向，准确解读相关政策，大力宣传两项保险合并实施的重要意义，让社会公众充分了解合并实施不会影响参保人员享受相关待遇，且有利于提高基金共济能力、减轻用人单位事务性负担、提高管理效率，为推动两项保险合并实施创造良好的社会氛围。

国务院办公厅
2019年3月6日
